# ‘Joke's A Part’: In defence of humour

**DOI:** 10.4103/0019-5545.31581

**Published:** 2006

**Authors:** G. Swaminath

**Affiliations:** * Professor and Head, Department of Psychiatry, Dr B.R. Ambedkar Medical College, Bangalore 560045; *mailing address:* Smruthy Clinic, 675, 11-A Main, 45 Cross, 3rd Block, Raja Jinaga, Bangalore 560010; e-mail: swamig@sify.com

Legend has it that shortly after Adam was created, he complained: ‘O, Lord! You have given the lion fierce teeth and claws, and the elephant formidable tusks; you have given the deer swiftness of legs, and the turtle a protective shell; you have given the birds of flight wings, but you have left me altogether defenceless.’ And the Lord said unto Adam: ‘I shall give you an invisible weapon that will serve you and your children better than any weapons of fight or flight, a power that will save you even from yourself. I shall give you the sense of humour.’

We are defenceless without humour. If we fail to see the irony in our circumstances, the situation may appear dispiriting. Laughter is a way of ‘thumbing one's nose’ at the inescapable and incomprehensible vagaries of existence and declaring, ‘I choose to rise above this. I choose to meet life head on.’ Laughter is freedom.

There is a mistaken belief that since the medical profession is critical, the doctor should appear intense, sincere and solemn, but definitely not flippant. Too many people confuse seriousness with professionalism and put a lid on the sense of humour. They think joking would render the doctor's behaviour unprofessional. Physicians tend to be wary of laughing with their patients. Our training enforces the idea that healing is a serious business. Doctors do not want to appear frivolous and flippant, especially with people they do not know.

## IS HUMOUR THERAPEUTIC?

As early as the 1300s, Henri de Mondeville, professor of surgery, propagated therapy with humour to patients after surgery ‘by having someone tell him jokes’. While there are several anecdotal reports claiming humour to be therapeutic, there are no double-blind control trials. In *Anatomy of an illness,* Norman Cousins (1976) first called the attention of the medical community to the potential therapeutic effects of humour when he described his utilization of laughter during treatment for his ankylosing spondylitis. Believing that negative emotions had a negative impact on his health, he theorized that positive emotions would have a positive effect. He believed that the experience of laughter could open him to feelings of joy, hope, confidence and love. ‘If you can laugh at it, you can survive it.’

‘Even if laughter produces no specific biochemical changes,’ according to Norman Cousins, ‘it accomplishes one very essential purpose. It tends to block deep feelings of apprehension and even panic that all too frequently accompany serious illness. It helps free the body of the constricting effects of the negative emotions that in turn may impair the healing system.’ According to Cousins, 10 minutes of laughter resulted in 2 hours of pain-free sleep.

### How does humour operate as therapy?

Sigmund Freud had a theory about humour which was a synthesis of the three theories of humour, i.e. Incongruity, relief and conflict theories. Another theory propounded is the superiority theory, e.g. when we laugh at someone who has been duped.

Freud postulated that humour works by means of two principal mechanisms, ‘condensation’ and ‘displacement’. Condensation entails an economy in thought and expression and conserves psychic energy, and displacement transfers this psychic energy arising from conflict or incongruity to a humorous anecdote, which brings relief. Freud believed that cultivating a sense of humour could help lift repressions (i.e. unconscious conflictual material) but could also be harmful, particularly in certain forms of sarcasm and irony, directed at the self.

Kahn identified five primary functions that humour serves for individuals and groups:

1. *Coping.* By using humour as a coping mechanism, people are able to bear the burden of suffering or misfortune. This is done by recognizing the incongruity of believing that one is the only person suffering, thus increasing one's sense of shared humanity.
2. *Reframing.* This involves looking at things in a different way, a form of cognitive reappraisal with affective elements.
3. *Communicating.* Humour can be an unobtrusive way of seeking help. If a person moans and groans continually to potential helpers, they are in fact less likely to help and will avoid the complainer. Using humour disarms their defences and also conveys the message that while help is needed, the person seeking help is essentially able to cope and will not place unbearable burden upon the helpers. Of course, this can also work in the negative sense-if humour is used to excessively play down problems, then friends may assume that no help is needed.
4. *Expressing hostility.* Humour, particularly sarcasm and irony can be very sophisticated expressions of anger and criticism and are very hard to answer, since an attack is conveyed in such a way as to circumvent logic and rational discussion. Again, this can have positive and negative consequences, depending upon the basic level of under-standing and trust operating between speaker and interlocutor. Sarcasm and irony directed at oneself can be very harmful.
5. *Constructing identities.* By expressing a sense of humour, a person gives out the message that they are fun-loving, easy-going and are good company. Too much humour however may result in people forming the judgement that they are immature and shallow. Once again a balance needs to be struck.

## HOW COULD HUMOUR BE OF HELP TO CLIENTS?

Humour on the part of the therapist can be of potential help to clients in a number of ways:

- It creates a more relaxed atmosphere and helps break down barriers.
- It can convey the message that the therapist is humane.
- It can build trust and empathy if used appropriately.
- It can help the client to relax and possibly lift repressions.
- It can convey messages succinctly and effectively.
- It encourages communication on sensitive matters.
- It can be a source of insight into conflict.
- It can help overcome a stiff and formal communicating style.
- It can facilitate the acting out of feelings or impulses in a safe, non-threatening way.

### Humour that enhances the counselling relationship

Humour on the part of the therapist can build and enhance the therapeutic relationship. At all times such humour should be good-natured, natural and directed at laughing with rather than at the client. Therapeutic use of humour can result in a client's putting problems into perspective. Counsellors can help clients learn to take themselves less seriously and even laugh at some of the foolishness of their behaviour.

Clients and counsellors can enrich a relationship by laughing. Humour and tragedy are closely linked and after allowing ourselves to feel some experiences that are painfully tragic, we can also genuinely laugh at how seriously we have taken our situation. We secretly delude ourselves into believing that we are unique in that we are alone in our pain and we alone have experienced the tragic. What a welcome relief when we can admit that pain is not our exclusive domain.

### Humour that detracts from the counselling relationship

There are times, of course, when laughter is used to cover up anxiety or to escape from the experience of facing threatening material. The therapist needs to distinguish between humour that distracts and humour that enhances the situation. Humour which belittles, embarrasses or intimidates the client is to be avoided at all costs. Clients also sometimes use humour as a defence to avoid feeling the pain of difficult material or as a distraction. This needs to be worked through by the therapist.

### Humour as a defence mechanism

Humour can be used by clients to defend against painful material or as a distracting device to change the topic when painful material is covered. The therapist needs to assess whether this is a healthy or unhealthy defence. It may be that the client is not yet ready to work with the painful stuff, or is ready but is unwilling to do the work. Here the therapist could point out that the client has used humour to change the topic and go on to explore the reasons for this.

Several practitioners have written about the role that fun and humour have in psychotherapy and counselling. To be effective, humour must be timed appropriately. It is usually unwise to employ humour until the therapeutic relationship has been well established. Once this involvement with a client exists, it is far more likely that humour will result in a positive outcome. Therapeutic humour has an educative, corrective message, and it helps clients put situations in perspective. Such humour does not involve hostility, ridicule, or a lack of respect. On the other hand, harmful humour exacerbates a client's problems, undermines a sense of personal worth, and leaves the person feeling resentful. It goes without saying that humour that undermines the self-esteem of clients by deprecating and humiliating them is inappropriate, and works against therapeutic aims.

## DO MANY PSYCHIATRISTS SUFFER FROM A LAUGHTER DEFICIENCY DISORDER?

Unlike the doctor–patient relationship in the medical setting, the psychiatrist–client relationship seems to start with an inherent disadvantage. Psychiatrists develop skills that help people to cope with their mental health problems, enabling them to make progress towards a solution after other help has failed. People with mental illness are extremely unhappy and difficult to reach; they may feel cut off from the rest of the world and find it almost impossible to have trust or confidence in anyone. The psychiatrist can be the person who can make a difference and can give hope at the most despairing times. While cures are often difficult to effect, psychiatrists can make an enormous contribution to improving the quality of life of their patients, reducing their symptoms and distress, and making an impact on their social conditions. A good psychiatrist must find a workable balance between the toughness necessary to face up to difficult and even threatening behaviour on a regular basis, while at the same time retaining sensitivity, compassion, and interpersonal skills. Such a career is by no means easy, but the rewards can be great.

Humour is not necessarily telling jokes or comedy. It is a sense of delight and exuberance that life is funny. In an article in the *BMJ,* Anne Dean analyses the characteristics necessary for a successful career in psychiatry and lists the following:

- Varied life experiences before entry to the specialty
- Interests such as travel, theatre, music or the arts
- Intellectual rigour combined with all round ability
- An inquisitive and humane nature
- A well developed sense of humour
- Excellent communication and interpersonal skills
- A calm, unflappable nature
- Patience, tact, and the ability to work with uncertainty,

In a specialty where there are often no easy answers, many patients must be managed for a very long time. Psychiatry is not for doctors who thrive on immediate results or the excitement of making a split second decision—though there may be occasions when this is necessary. It is here that a sense of humour helps us to keep things in perspective and not take ourselves too seriously. It is said ‘Imagination was given to a man to compensate him for what he is not; a sense of humour was provided to console him for what he is.’ Laughter can take us from moping, to coping, to hoping.

## HUMOUR IN THERAPY

The use of humour as a therapeutic technique has been advocated by Ellis *et al.* Others have also advocated the use of humour on the path towards personal growth. Rational Emotive Therapy (RET) counsellors tend to be appropriately humorous with their clients since much emotional disturbance stems from clients taking themselves, their problems, other people and the world too seriously. ‘Counsellors do not poke fun at the clients themselves but at their self-defeating thoughts, feelings and actions’ and that the purpose of using humour in this way is to ‘strive to model for their clients the therapeutic advantages of taking a serious but humorously ironic attitude to life’. This is achieved by using techniques such as: exaggeration, paradox, humorous songs, etc. An example is given of how the therapist in trying to help a client, makes an exaggerated display of concern, by treating her as if she could hardly walk and by escorting her by the arm to the chair. This eventually prompted the client to respond with ‘Don't treat me like a child’ and ‘I can cope’, a response which could never have been achieved by a more directly didactic approach.

However, it should be noted that the therapist could be so easily led into abusing humour as a powerful tool to ‘educate’ the client. Also one must take care to avoid giving a message to the client that, in addition to having psychological problems, they are also stupid and need to be treated like children.

## SNAPSHOTS FROM MY CONSULTING ROOM

Humour commonly offends somebody. Whether we laugh at someone or with someone is equally dependent on the context of the relationship between the two. For example even if we laugh at ourselves, our present self feels superior and laughs at the incongruity of thought or behaviour and foolishness of our past self. A skill is required to wield that weapon of humour without appearing insensitive or downright manic. As healers we should be mindful of cultural sensitivities and other bigotry. On the other hand, I may have inadvertently caused the patient to leave the practice, thinking I was offensive.

Most of the laughter and joy in our life comes from everyday experiences. Slips of the tongue, puns, incongruities, overheard conversations, bureaucratic foul-ups, and silly personal mistakes make us laugh every day.

It is the spontaneous use of the same ‘opportunities’ to generate laughter, which enlivens my practice and probably helps in improving relationship with clients and puts across messages effectively. Humour could also be used to convey messages effectively. The main message has a smooth piggy-back on humour. Many of the important issues to be discussed with patients, like the prolonged duration of treatment, latency of action of drugs, side effects of drugs, dosage of drugs and number of tablets, paying for the ‘talking cure’, etc. could be tackled effectively with humour. While explaining a treatment plan to patients, levity can be used to gain cooperation.

Many a time doctors fumble over the opening line. I usually set the humorous tone from the beginning itself. Probably my ability to use unconventional terms and street lingo in Kannada not expected of a doctor, is a source of amusement. A cheery ‘Come Boss’, ‘I think you should eat more and gain strength to close the door, it is tight’, or a smile and a ‘What Ya?’ to a child sets the tone right for the interview. Opportunities for setting the humorous tone could arise even when there is a telephone conversation for first appointment or one for clarifications. This usually reduces anxiety and you could almost visualize them chuckling. These people have never interacted with me and have a sense of discomfort that makes them either formal or diffident. Use of humour in this instance could break the ice. If they ask me when the clinic opens I say ‘The clock rings nine times at nine. I open with the first dong.’

Some patients tell me that they would like to speak to me when I am totally free, I say, ‘I pray to god everyday that I am not free, but you can come anytime.’ Each opportunity can provide an occasion for humour. Use of radio, film, TV banter and one liners, incongruous in doctor patient setting also amuses the patient and sets a humorous tone.

### Sarcasm

Usually mockery, ridicule, and sarcasm have no place in a healing relationship. Laughter should restore a person's dignity and self-worth, not destroy it. Humour must be appropriate and benevolent. But this is not always the case. Our patients too realize this especially when they understand our concern. However, tone is very important in handing out sarcasm. I use many comments which could be considered inappropriate when spoken by others. I once told a complaining auditor patient, ‘You put an audit objection to whatever is advised.’ He went back laughing and would quote my comment to help me identify him over the phone. Other examples include:

- ‘What I have given you is treatment not punishment.’
- ‘If I work on Sundays I too will have to go to see another doctor.’
- ‘If you do not follow advice, then with repeated consul-tations, I should be able to complete the renovation of my house.’

Though occasionally we may go overboard and hurt other's sentiments, all is not lost. If you become aware, backtracking with the use of a disarming smile or an apology can smoothen things.

Sometimes humour is very effective to cut short a long winding history, which has many repetitions, or to terminate an interview wherein patients are nagging you to commit yourself where you do not want to e.g. advise marriage, or, issue a certificate.

## CONCLUSION

Looking back, I see that I am enjoying my practice. Humour makes it less dreary. But I am not sure if it has been the same for my patients. I am certain that many of them have felt that I was too frivolous for consumption. Instead of being comfortable, they could have been offended. I have been cautioned by various friends in the profession of such incidents and this put me on leash at least temporarily. But my personality comes through again. Over time I have realized that I could change the manner in which I interact with my patients, and be more casual only with the people who accept my brand of humour. I have learnt the hard way that ‘dead pan’ humour does not go well with patients as it would with friends and colleagues. I have learnt to smile immediately after a joke if I see that it has missed the patient by a mile in order to reassure them that it was but a joke.

John F. Kennedy wrote, ‘There are three things which are real: God, human folly, and laughter. The first two are beyond comprehension. So we must do what we can with the third.’ The pursuit of happiness is one of life's basic precepts. Patch Adams, a physician who is also a clown, believes that humour and love are at the core of good bedside manner, preventing burnout, and avoiding malpractice. Humour deserves a special place in medical practice.

‘If you want to rule the world, keep it amused,’ said Emerson. I do not want to rule the world but I definitely want to keep it amused and amuse myself in the bargain. ‘Humour is this psychiatrist's visiting card.’ I beseech that professional brethren try using it too.
